# (*E*)-3-Chloro-*N*′-hy­droxy­benzene-1-carboximidamide

**DOI:** 10.1107/S1600536812046612

**Published:** 2012-11-24

**Authors:** S. Sreenivasa, K. E. ManojKumar, P. A. Suchetan, N. R Mohan, B. S. Palakshamurthy

**Affiliations:** a Department of Studies and Research in Chemistry, Tumkur University, Tumkur, Karnataka 572 103, India; b Department of Studies and Research in Chemistry, U.C.S, Tumkur University, Tumkur, Karnataka 572 103, India; c Department of Studies and Research in Physics, U.C.S., Tumkur University, Tumkur, Karnataka 572 103, India

## Abstract

The title compound, C_7_H_7_ClN_2_O, crystallizes with two independent mol­ecules in the asymmetric unit. The compound adopts an *E* configuration across the C=N double bond, as the –OH group and the benzene ring are on opposite sides of the double bond while the H atom of the hy­droxy group is directed away from the –NH_2_ group. In the crystal, mol­ecules are linked to one another through O—H⋯N and N—H⋯O hydrogen bonds, forming chains along [010].

## Related literature

For related syntheses and the biological activity of oxadiazo­les, see: Kundu *et al.* (2012 ▶); Sakamoto *et al.* (2007 ▶); Tyrkov & Sukhenko (2004 ▶). For hydrogen-bond motifs, see: Bernstein *et al.* (1995 ▶).
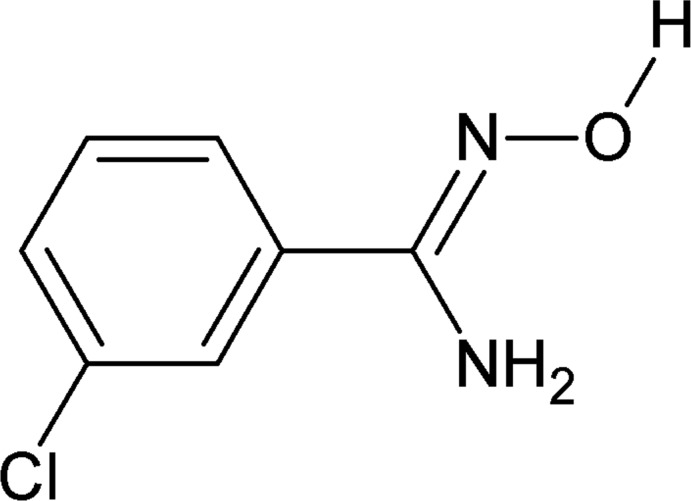


## Experimental

### 

#### Crystal data

C_7_H_7_ClN_2_O
*M* *_r_* = 170.60
Triclinic, 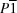

*a* = 5.0018 (17) Å
*b* = 10.984 (4) Å
*c* = 14.407 (6) Å
α = 74.000 (12)°
β = 89.952 (12)°
γ = 89.877 (11)°
*V* = 760.9 (5) Å^3^
*Z* = 4
Mo *K*α radiation
μ = 0.44 mm^−1^
*T* = 298 K
0.24 × 0.22 × 0.20 mm

#### Data collection

Bruker SMART X2S diffractometer
Absorption correction: multi-scan (*SADABS*; Sheldrick, 2004 ▶) *T*_min_ = 0.902, *T*_max_ = 0.917
14972 measured reflections
2655 independent reflections
2192 reflections with *I* > 2σ(*I*)
*R*_int_ = 0.060

#### Refinement

*R*[*F* ^2^ > 2σ(*F* ^2^)] = 0.037
*wR*(*F* ^2^) = 0.109
*S* = 1.05
2655 reflections
218 parameters
H atoms treated by a mixture of independent and constrained refinement
Δρ_max_ = 0.19 e Å^−3^
Δρ_min_ = −0.24 e Å^−3^

### 

Data collection: *APEX2* (Bruker, 2004 ▶); cell refinement: *APEX2* and *SAINT-Plus* (Bruker, 2004 ▶); data reduction: *SAINT-Plus* and *XPREP* (Bruker, 2004 ▶); program(s) used to solve structure: *SHELXS97* (Sheldrick, 2008 ▶); program(s) used to refine structure: *SHELXL97* (Sheldrick, 2008 ▶); molecular graphics: *ORTEP-3* (Farrugia, 2012 ▶); software used to prepare material for publication: *SHELXL97*.

## Supplementary Material

Crystal structure: contains datablock(s) I, global. DOI: 10.1107/S1600536812046612/jj2159sup1.cif

Structure factors: contains datablock(s) I. DOI: 10.1107/S1600536812046612/jj2159Isup2.hkl

Supplementary material file. DOI: 10.1107/S1600536812046612/jj2159Isup3.cml

Additional supplementary materials:  crystallographic information; 3D view; checkCIF report

## Figures and Tables

**Table 1:** Hydrogen-bond geometry (Å, °)

*D*—H⋯*A*	*D*—H	H⋯*A*	*D*⋯*A*	*D*—H⋯*A*
O2—H2*A*⋯N4^i^	0.82	2.09	2.811 (2)	147
N3—H3*B*⋯O1^ii^	0.92 (3)	2.32 (3)	3.006 (2)	131 (2)
O1—H1⋯N2^iii^	0.82	2.08	2.805 (2)	147
N1—H1*B*⋯O2^iv^	0.87 (3)	2.36 (3)	3.006 (3)	132 (3)

Symmetry codes: (i) 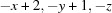; (ii) 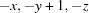; (iii) 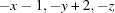; (iv) 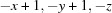.

**Fig. 1.**
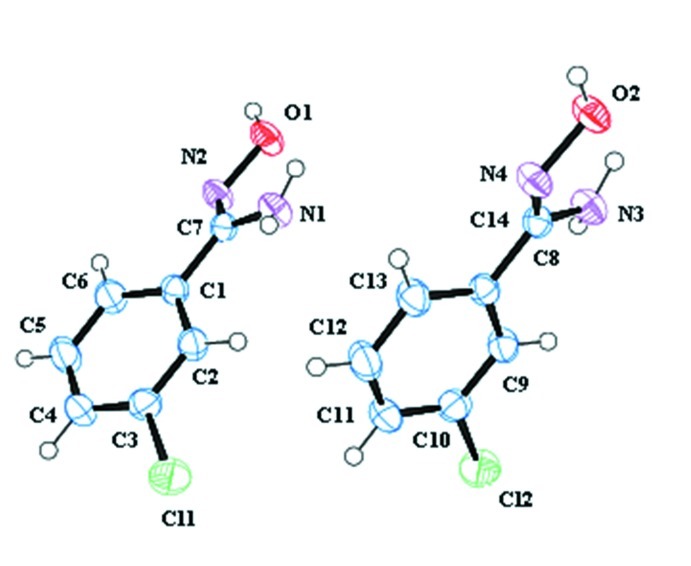
Molecular structure of the title compound, showing the atom-labeling scheme. Displacement ellipsoids are drawn at the 50% probability level.

**Fig. 2.**
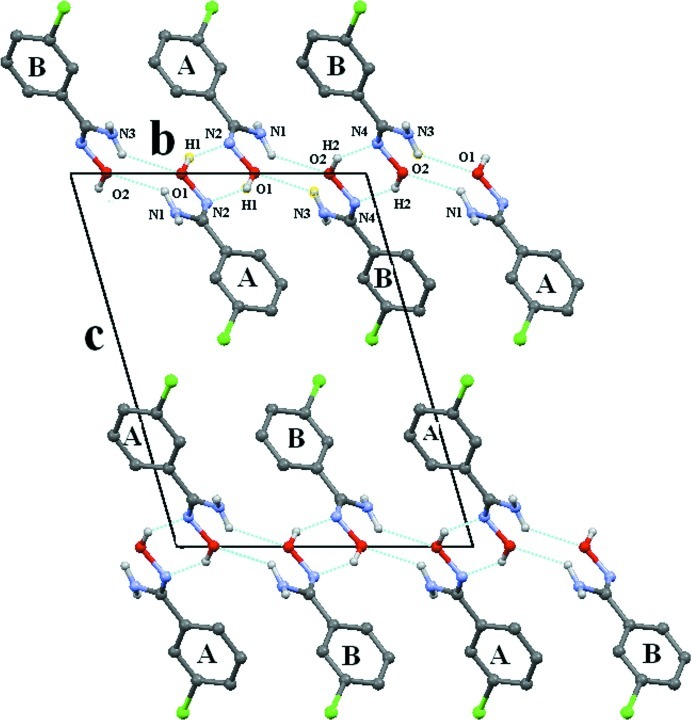
Molecular packing of the title compound when viewed along the a axis. O—H···N and N—H···O hydrogen bonds are shown as dashed lines. Hydrogen atoms not involved in hydrogen bonding are omitted for clarity.

## supplementary crystallographic information

### Comment

Substituted *N*'-hydroxybenzamidines is a key intermediate obtained during the synthesis of pharmaceutically important 1,2,4-oxadiazole derivatives. 1,2,4-Oxadiazole derivatives are well known for their anti-HIV and anti-microbial activities (Kundu *et al.*, 2012; Sakamoto *et al.*, 2007; Tyrkov *et al.*, 2004). In our studies on these types of compounds, we synthesized the title compound,C_7_H_7_ClN_2_O, (I) and report here its crystal structure.

The title compound, (I), crystallizes with two independent molecules (A & B) in the asymmetric unit (Fig. 1). The compound prefers an E configuration across the C—N double bond, as the OH group and the benzene ring are on opposite sides of the double bond while the hydrogen atom of the hydroxyl group is directed away from the NH_2_ group. In the crystal, the independent molecules (A & B) are connected to their respective crystallographically identical molecules through O1—H1···N2 and O2—H2···N4 intermolecular hydrogen bonds, each forming *R*_2_^2^(6) dimeric pairs (Fig 2). The dimeric pairs are further connected to one another through intermolecular N1-H1N1···O2 and N3-H3N3···O1 hydrogen bonds forming a chain of ring patterns (Bernstein *et al.*, 1995) along [010] (Fig 2).

### Experimental

To a solution of 3-chlorobenzonitrile (1 mmol) in ethanol was added triethyl amine (2.5 mmol) and NH_2_OH.HCl (3.5 mmol). The reaction mixture was stirred at room temperature for 12hrs. (The reaction was monitored by TLC). The solvent was removed and the crude product was purified by column chromatography using hexane and ethyl acetate as the eluent. Single crystals required for X-ray diffraction measurements were obtained from slow evaporation of the solution of the compound in a mixture of ethanol and dichloromethane (1:4).

### Refinement

The hydrogen atoms attached to N and O were located in difference maps and refined isotropically. The remaining H atoms were positioned geometrically and refined using a riding model, with C–H = 0.93 Å with isotropic displacement parameters set to 1.2 times of the *U*_eq_ of the parent atom.

### Crystal data

**Table:**

C_7_H_7_ClN_2_O	*F*(000) = 352
*M**_r_* = 170.60	Prism
Triclinic, *P*1	*D*_x_ = 1.489 Mg m^−^^3^
Hall symbol: -P 1	Melting point: 386 K
*a* = 5.0018 (17) Å	Mo *K*α radiation, λ = 0.71073 Å
*b* = 10.984 (4) Å	Cell parameters from 218 reflections
*c* = 14.407 (6) Å	θ = 1.9–25°
α = 74.000 (12)°	µ = 0.44 mm^−^^1^
β = 89.952 (12)°	*T* = 298 K
γ = 89.877 (11)°	Prism, colourless
*V* = 760.9 (5) Å^3^	0.24 × 0.22 × 0.20 mm
*Z* = 4	

### Data collection

**Table:**

Bruker SMART X2S diffractometer	2655 independent reflections
Radiation source: fine-focus sealed tube	2192 reflections with *I* > 2σ(*I*)
Graphite monochromator	*R*_int_ = 0.060
Detector resolution: 1.20 pixels mm^-1^	θ_max_ = 25.0°, θ_min_ = 1.9°
phi and ω scans	*h* = −5→5
Absorption correction: multi-scan (*SADABS*; Sheldrick, 2004)	*k* = −13→13
*T*_min_ = 0.902, *T*_max_ = 0.917	*l* = −17→17
14972 measured reflections	

### Refinement

**Table:**

Refinement on *F*^2^	Secondary atom site location: difference Fourier map
Least-squares matrix: full	Hydrogen site location: inferred from neighbouring sites
*R*[*F*^2^ > 2σ(*F*^2^)] = 0.037	H atoms treated by a mixture of independent and constrained refinement
*wR*(*F*^2^) = 0.109	*w* = 1/[σ^2^(*F*_o_^2^) + (0.0546*P*)^2^ + 0.1852*P*] where *P* = (*F*_o_^2^ + 2*F*_c_^2^)/3
*S* = 1.05	(Δ/σ)_max_ = 0.001
2655 reflections	Δρ_max_ = 0.19 e Å^−^^3^
218 parameters	Δρ_min_ = −0.24 e Å^−^^3^
0 restraints	Extinction correction: *SHELXL97* (Sheldrick, 2008), Fc^*^=kFc[1+0.001xFc^2^λ^3^/sin(2θ)]^-1/4^
0 constraints	Extinction coefficient: 0.024 (4)
Primary atom site location: structure-invariant direct methods	

### Special details

**Table:**

Geometry. All e.s.d.'s (except the e.s.d. in the dihedral angle between two l.s. planes) are estimated using the full covariance matrix. The cell e.s.d.'s are taken into account individually in the estimation of e.s.d.'s in distances, angles and torsion angles; correlations between e.s.d.'s in cell parameters are only used when they are defined by crystal symmetry. An approximate (isotropic) treatment of cell e.s.d.'s is used for estimating e.s.d.'s involving l.s. planes.
Refinement. Refinement of *F*^2^ against ALL reflections. The weighted *R*-factor *wR* and goodness of fit *S* are based on *F*^2^, conventional *R*-factors *R* are based on *F*, with *F* set to zero for negative *F*^2^. The threshold expression of *F*^2^ > σ(*F*^2^) is used only for calculating *R*-factors(gt) *etc*. and is not relevant to the choice of reflections for refinement. *R*-factors based on *F*^2^ are statistically about twice as large as those based on *F*, and *R*- factors based on ALL data will be even larger.

### Fractional atomic coordinates and isotropic or equivalent isotropic displacement parameters (Å^2^)

**Table:**

	*x*	*y*	*z*	*U*_iso_*/*U*_eq_	
H1B	0.079 (5)	0.805 (3)	0.050 (2)	0.066 (8)*	
H3B	0.430 (5)	0.301 (3)	0.047 (2)	0.065 (8)*	
H3A	0.247 (5)	0.322 (2)	0.1254 (17)	0.043 (6)*	
H1A	0.256 (5)	0.823 (2)	0.124 (2)	0.060 (7)*	
Cl1	0.42873 (11)	0.85954 (5)	0.44393 (4)	0.0554 (2)	
O1	−0.3214 (3)	0.87635 (13)	0.00143 (11)	0.0422 (4)	
H1	−0.4285	0.9158	−0.0388	0.063*	
N1	0.0928 (3)	0.81988 (15)	0.10588 (14)	0.0382 (4)	
N2	−0.3071 (3)	0.93253 (14)	0.08000 (12)	0.0356 (4)	
C1	−0.0344 (3)	0.95168 (15)	0.20990 (13)	0.0300 (4)	
C2	0.1471 (3)	0.89050 (16)	0.28068 (14)	0.0345 (4)	
H2	0.2326	0.8170	0.2763	0.041*	
C3	0.1997 (4)	0.93902 (17)	0.35722 (14)	0.0363 (4)	
C4	0.0746 (4)	1.04675 (18)	0.36709 (15)	0.0417 (5)	
H4	0.1116	1.0783	0.4193	0.050*	
C5	−0.1068 (4)	1.10622 (19)	0.29740 (16)	0.0456 (5)	
H5	−0.1939	1.1787	0.3030	0.055*	
C6	−0.1621 (4)	1.06053 (17)	0.21941 (15)	0.0398 (5)	
H6	−0.2849	1.1024	0.1731	0.048*	
C7	−0.0862 (3)	0.90061 (15)	0.12633 (13)	0.0285 (4)	
Cl2	0.07127 (12)	0.35954 (5)	0.44389 (4)	0.0556 (2)	
O2	0.8210 (3)	0.37629 (13)	0.00125 (11)	0.0420 (3)	
H2A	0.9256	0.4165	−0.0396	0.063*	
N3	0.4070 (4)	0.31958 (15)	0.10577 (14)	0.0381 (4)	
N4	0.8071 (3)	0.43247 (15)	0.08013 (12)	0.0357 (4)	
C8	0.5338 (3)	0.45141 (15)	0.20995 (13)	0.0298 (4)	
C9	0.3535 (4)	0.39047 (16)	0.28028 (14)	0.0350 (4)	
H9	0.2684	0.3172	0.2757	0.042*	
C10	0.3003 (4)	0.43874 (17)	0.35716 (14)	0.0363 (4)	
C11	0.4257 (4)	0.54666 (18)	0.36691 (15)	0.0422 (5)	
H11	0.3885	0.5782	0.4192	0.051*	
C12	0.6065 (4)	0.60617 (19)	0.29736 (16)	0.0457 (5)	
H12	0.6931	0.6786	0.3030	0.055*	
C13	0.6620 (4)	0.56053 (17)	0.21931 (15)	0.0396 (5)	
H13	0.7845	0.6023	0.1729	0.048*	
C14	0.5860 (3)	0.40097 (15)	0.12632 (13)	0.0286 (4)	

### Atomic displacement parameters (Å^2^)

**Table:**

	*U*^11^	*U*^22^	*U*^33^	*U*^12^	*U*^13^	*U*^23^
Cl1	0.0630 (4)	0.0547 (3)	0.0467 (4)	0.0016 (2)	−0.0246 (3)	−0.0106 (2)
O1	0.0441 (8)	0.0484 (8)	0.0418 (8)	0.0024 (6)	−0.0111 (6)	−0.0251 (7)
N1	0.0349 (10)	0.0440 (9)	0.0412 (10)	0.0039 (7)	−0.0025 (7)	−0.0210 (8)
N2	0.0357 (9)	0.0405 (8)	0.0357 (9)	0.0016 (6)	−0.0075 (7)	−0.0191 (7)
C1	0.0289 (9)	0.0297 (8)	0.0321 (10)	−0.0037 (6)	−0.0001 (7)	−0.0097 (7)
C2	0.0353 (10)	0.0311 (9)	0.0383 (11)	0.0006 (7)	−0.0048 (8)	−0.0114 (8)
C3	0.0358 (10)	0.0381 (9)	0.0335 (10)	−0.0059 (7)	−0.0049 (8)	−0.0075 (8)
C4	0.0522 (12)	0.0401 (10)	0.0375 (11)	−0.0075 (8)	−0.0013 (9)	−0.0185 (9)
C5	0.0544 (13)	0.0391 (10)	0.0500 (13)	0.0068 (8)	−0.0044 (10)	−0.0233 (9)
C6	0.0418 (11)	0.0377 (10)	0.0417 (11)	0.0078 (8)	−0.0107 (8)	−0.0138 (8)
C7	0.0284 (9)	0.0263 (8)	0.0308 (9)	−0.0042 (6)	0.0003 (7)	−0.0081 (7)
Cl2	0.0633 (4)	0.0552 (3)	0.0465 (4)	−0.0023 (2)	0.0241 (3)	−0.0109 (2)
O2	0.0445 (8)	0.0488 (8)	0.0404 (8)	−0.0033 (6)	0.0107 (6)	−0.0252 (7)
N3	0.0331 (10)	0.0442 (9)	0.0423 (10)	−0.0049 (7)	0.0039 (7)	−0.0211 (8)
N4	0.0347 (9)	0.0411 (8)	0.0367 (9)	−0.0018 (6)	0.0056 (7)	−0.0197 (7)
C8	0.0290 (9)	0.0296 (8)	0.0320 (10)	0.0029 (6)	0.0001 (7)	−0.0103 (7)
C9	0.0351 (10)	0.0313 (9)	0.0401 (11)	−0.0006 (7)	0.0044 (8)	−0.0122 (8)
C10	0.0374 (11)	0.0366 (9)	0.0334 (10)	0.0054 (7)	0.0049 (8)	−0.0074 (8)
C11	0.0538 (13)	0.0404 (10)	0.0366 (11)	0.0076 (8)	0.0004 (9)	−0.0181 (9)
C12	0.0545 (13)	0.0390 (10)	0.0497 (13)	−0.0072 (9)	0.0040 (10)	−0.0225 (9)
C13	0.0423 (11)	0.0373 (10)	0.0420 (12)	−0.0085 (8)	0.0083 (8)	−0.0154 (8)
C14	0.0273 (9)	0.0274 (8)	0.0311 (9)	0.0035 (6)	−0.0005 (7)	−0.0081 (7)

### Geometric parameters (Å, º)

**Table:**

Cl1—C3	1.7412 (19)	Cl2—C10	1.742 (2)
O1—N2	1.433 (2)	O2—N4	1.436 (2)
O1—H1	0.8200	O2—H2A	0.8200
N1—C7	1.347 (2)	N3—C14	1.356 (2)
N1—H1B	0.87 (3)	N3—H3B	0.92 (3)
N1—H1A	0.86 (3)	N3—H3A	0.85 (2)
N2—C7	1.288 (2)	N4—C14	1.288 (2)
C1—C2	1.391 (2)	C8—C9	1.384 (3)
C1—C6	1.394 (3)	C8—C13	1.399 (2)
C1—C7	1.485 (2)	C8—C14	1.481 (2)
C2—C3	1.376 (3)	C9—C10	1.379 (3)
C2—H2	0.9300	C9—H9	0.9300
C3—C4	1.379 (3)	C10—C11	1.383 (3)
C4—C5	1.377 (3)	C11—C12	1.375 (3)
C4—H4	0.9300	C11—H11	0.9300
C5—C6	1.380 (3)	C12—C13	1.379 (3)
C5—H5	0.9300	C12—H12	0.9300
C6—H6	0.9300	C13—H13	0.9300
			
N2—O1—H1	109.5	N4—O2—H2A	109.5
C7—N1—H1B	116.7 (17)	C14—N3—H3B	116.1 (16)
C7—N1—H1A	118.6 (17)	C14—N3—H3A	117.6 (15)
H1B—N1—H1A	114 (2)	H3B—N3—H3A	117 (2)
C7—N2—O1	109.78 (14)	C14—N4—O2	109.65 (14)
C2—C1—C6	118.65 (17)	C9—C8—C13	118.93 (17)
C2—C1—C7	119.67 (15)	C9—C8—C14	119.71 (15)
C6—C1—C7	121.67 (16)	C13—C8—C14	121.36 (16)
C3—C2—C1	119.83 (17)	C10—C9—C8	119.82 (16)
C3—C2—H2	120.1	C10—C9—H9	120.1
C1—C2—H2	120.1	C8—C9—H9	120.1
C2—C3—C4	121.88 (17)	C9—C10—C11	121.64 (18)
C2—C3—Cl1	118.24 (14)	C9—C10—Cl2	118.40 (14)
C4—C3—Cl1	119.87 (15)	C11—C10—Cl2	119.96 (15)
C5—C4—C3	118.08 (18)	C12—C11—C10	118.32 (18)
C5—C4—H4	121.0	C12—C11—H11	120.8
C3—C4—H4	121.0	C10—C11—H11	120.8
C4—C5—C6	121.40 (18)	C11—C12—C13	121.28 (18)
C4—C5—H5	119.3	C11—C12—H12	119.4
C6—C5—H5	119.3	C13—C12—H12	119.4
C5—C6—C1	120.15 (18)	C12—C13—C8	120.01 (18)
C5—C6—H6	119.9	C12—C13—H13	120.0
C1—C6—H6	119.9	C8—C13—H13	120.0
N2—C7—N1	123.84 (17)	N4—C14—N3	123.71 (17)
N2—C7—C1	117.48 (15)	N4—C14—C8	117.58 (15)
N1—C7—C1	118.61 (16)	N3—C14—C8	118.62 (16)

### Hydrogen-bond geometry (Å, º)

**Table:**

*D*—H···*A*	*D*—H	H···*A*	*D*···*A*	*D*—H···*A*
O2—H2*A*···N4^i^	0.82	2.09	2.811 (2)	147
N3—H3*B*···O1^ii^	0.92 (3)	2.32 (3)	3.006 (2)	131 (2)
O1—H1···N2^iii^	0.82	2.08	2.805 (2)	147
N1—H1*B*···O2^iv^	0.87 (3)	2.36 (3)	3.006 (3)	132 (3)

Symmetry codes: (i) −*x*+2, −*y*+1, −*z*; (ii) −*x*, −*y*+1, −*z*; (iii) −*x*−1, −*y*+2, −*z*; (iv) −*x*+1, −*y*+1, −*z*.
